# A Survey of Hospital Pharmacy Guidelines for the Administration of 3% Sodium Chloride in Children

**DOI:** 10.3390/children9010057

**Published:** 2022-01-03

**Authors:** Siddharth A. Shah, Juan C. Ayus, Michael L. Moritz

**Affiliations:** 1 Department of Pediatrics, Norton Children’s Hospital, University of Louisville, Louisville, KY 40202, USA; Siddharth.shah@louisville.com; 2 Division of Nephrology and Hypertension and Kidney Transplantation, University of California Irvine, Orange, CA 92617, USA; Carlosayus@yahoo.com; 3 Division of Nephrology, Department of Pediatrics, University of Pittsburgh School of Medicine, Pittsburgh, PA 15213, USA; 4 Division of Nephrology, UPMC Children’s Hospital of Pittsburgh, Pittsburgh, PA 15224, USA

**Keywords:** hypertonic saline, 3% sodium chloride, hyponatremia, sodium, encephalopathy, children

## Abstract

Three percent sodium chloride (3% NaCl) is the treatment of choice for symptomatic hyponatremia. A barrier to the use of 3% NaCl is the perceived risk of both local infusion reactions and neurologic complications from overcorrection. We examine whether children’s hospital pharmacies have policies or practice guidelines for the administration of 3% NaCl and whether these pharmacies have restrictions on the administration of 3% NaCl in terms of rate, route, volume and setting. An Internet survey was distributed to the pharmacy directors of 43 children’s hospitals participating in the Children’s Hospital Association (CHA) network. The response rate was 65% (28/43). Ninety-three percent (26/28) of pharmacy directors reported a restriction for the administration of 3% NaCl, with 57% restricting its use through a peripheral vein or in a non-intensive care unit setting, 68% restricting the rate of administration and 54% restricting the volume of administration. Seventy-one percent (20/28) reported having written policy or practice guidelines. Only 32% of hospital pharmacies allowed 3% NaCl to be administered through a peripheral IV in a non-intensive care unit setting. The majority of children’s hospital pharmacies have restrictions on the administration of 3% NaCl. These restrictions could prevent the timely administration of 3% NaCl in children with symptomatic hyponatremia.

## 1. Introduction

Three-percent sodium chloride (3% NaCl, Na 513 mEq/L, 1027 mOsm/L) is a hyperosmolar agent primarily indicated for the treatment of hyponatremic encephalopathy or to raise the serum osmolality in other cases of increased intracranial pressure [1,2]. Barriers to the use of 3% NaCl include the perceived risk of a local infusion reaction when administered through a peripheral vein and the potential for complications from overcorrection of hyponatremia [3,4]. While local infusion reactions through a peripheral vein may occur with high-concentration total parenteral nutrition, potassium, or calcium, or 24% NaCl, this has not been reported with 3% NaCl [5,6]. Overcorrection of chronic hyponatremia has been implicated in the development of cerebral demyelination, also called osmotic demyelination syndrome [7,8]. The main reason for this complication is a spontaneous free-water diuresis leading to overcorrection, independent of receiving 3% NaCl [9].

We introduced the concept of using intermittent 3% NaCl boluses (2 mL/kg with a maximum of 100 mL) over 10 min with a goal of increasing the serum sodium by 5–6 mEq/L for the treatment of symptomatic hyponatremia [10,11]. This approach allows for a controlled and rapid increase in serum sodium to reduce cerebral edema, while at the same time minimizing the possibility for overcorrection. This approach can be used safely through a peripheral vein by emergency personnel in the outpatient setting, such as at high-endurance sporting events [12]. The 3% NaCl bolus, given with a goal of increasing the serum sodium by approximately 5 mEq/L, has been accepted as the preferred treatment for hyponatremic encephalopathy by experts in the field and is included in the European Clinical Practice Guidelines [2,13,14]. Recent clinical trials have demonstrated the superior efficacy of an intermittent bolus compared to a continuous infusion of 3% NaCl in achieving a more rapid increase in serum sodium when administered through a peripheral vein for the treatment of hyponatremic encephalopathy [15,16]. We have encountered situations in both children and adults where treating physicians were prohibited from administering 3% NaCl through a peripheral vein or in an acute care setting for the treatment of hyponatremia due to restrictive pharmacy policies. We therefore suspect that many hospital pharmacies may have established policies or practice guidelines restricting the use of 3% NaCl to avoid potential iatrogenic complications. These policies or guidelines may unintentionally restrict appropriate access to this potentially lifesaving therapy. The purpose of this study is to evaluate whether children’s hospital pharmacies have policies or practice guidelines for the administration of 3% NaCl and whether these pharmacies restrict the administration of 3% NaCl in terms of rate, route, volume and setting.

## 2. Methods

Following IRB approval, an anonymous Internet survey was distributed in 2011 to the pharmacy directors of 43 children’s hospitals participating in the Children’s Hospital Association (CHA), formerly called the Child Health Care Corporation of America (CHCA) network. There were six yes-or-no questions regarding policy and practice guidelines and potential restrictions for the administration of 3% NaCl and one question each regarding whether policy and practice guidelines existed for the administration of hypertonic mannitol or sodium bicarbonate (Table 1). The Fisher’s exact test was used to compare the restrictions between pharmacies with and without policy guidelines.

## 3. Results

**Figure 1:**
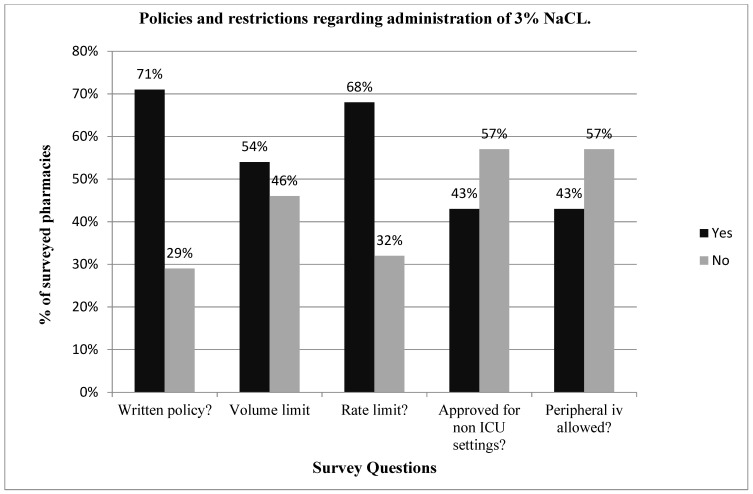
Reports on the policies and restrictions regarding the use of 3% NaCl in 38 major U.S. Children’s Hospital Pharmacies. A total of 71% of the pharmacies had written policies regarding the use of 3% NaCl, and the majority of hospital pharmacies imposed restrictions on the volume or rate of administration and do not allow administration through a peripheral vein.

**Figure 2:**
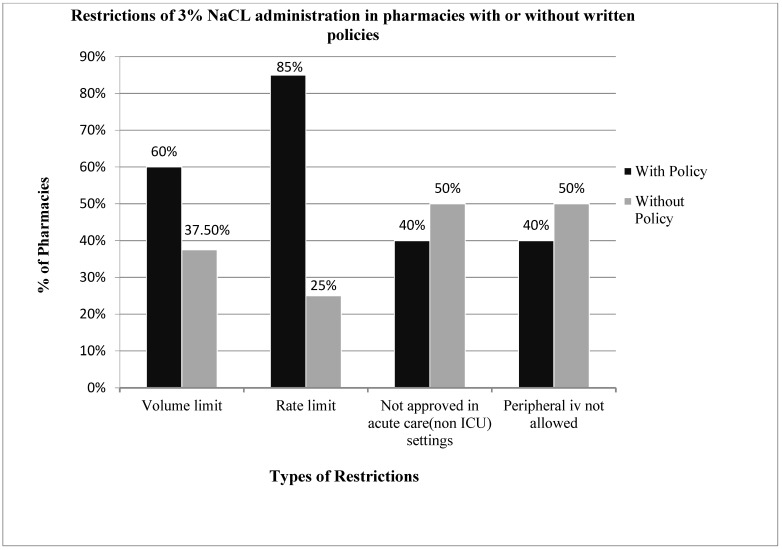
Compares the restrictions in administration of 3% sodium chloride among pharmacies with and without written policies. Hospital pharmacies with written policies were more likely to limit the volume and rate of administration. The requirements of ICU settings and central lines for administration were similar in pharmacies with and without written policies.

Twenty-eight pharmacy directors responded to the survey, giving a response rate of 65% (28/43). Overall, 93% (26/28) of the hospital pharmacy directors queried reported their institution’s pharmacy having some form of restriction for the administration of 3% NaCl, with 57% restricting its use through a peripheral vein or in a non-intensive care unit setting, 68% restricting the rate of administration and 54% restricting the volume of administration. Seventy-one percent (20/28) of hospital pharmacies had a written policy or practice guidelines for the administration of 3% NaCl (Figure 1), yet only 25% had written policies for the administration of hypertonic mannitol or sodium bicarbonate. Seventy-five percent (6/8) of pharmacies without written policies for 3% NaCl also had restrictions on the use of 3% NaCl (Figure 2), compared to 100% for those with written policies. Pharmacies with written policies were more likely to have restrictions on the volume and rate of administration (*p* < 0.05), but not on route or setting of administration (Figure 2). Only 32% (9/28) of hospital pharmacies allowed 3% NaCl to be administered through a peripheral IV in a non-ICU (inpatient floor) setting, and of those, 66% (6/9) had additional restrictions on the rate of administration. Only two pharmacies (7%) had no restrictions on the use of 3% NaCl.

## 4. Discussion

This survey demonstrates that most children’s hospital pharmacies have policies or practice guidelines which restrict the use of 3% NaCl. Even in pharmacies without explicit guidelines, the majority have restrictions on the use of 3% NaCl. The most concerning restrictions identified are restrictions which prohibit the use of 3% NaCl through a peripheral IV and in the non-ICU setting. Sixty-eight percent of children’s hospital pharmacies would not allow the use of 3% NaCl through a peripheral IV in a non-ICU setting. There are many reasons why these two policies are potentially dangerous. First and foremost is that it could result in an unnecessary delay of a potentially lifesaving therapy. Hyponatremic encephalopathy is a medical emergency that requires immediate therapy [17]. A patient with symptomatic hyponatremia could be suffering from increased intracranial pressure, and a delay in therapy could result in transtentorial herniation and death [18]. In addition, the placement of central venous access in a child is neither a benign nor a simple procedure. Sedation will likely be required to place a central line and this could be associated with either hypoxia or hypotension, both of which could aggravate hyponatremic encephalopathy by impairing brain cell volume regulation and decreasing cerebral perfusion [19]. Placement of a central line subjects the patient to the unnecessary risk of a central line infection, vascular thrombosis and long-term vascular stenosis [20]. A transfer to the ICU and placement of central line could delay therapy, result in increased costs and length of hospital stay, and could limit the access to an ICU bed of a patient that is more in need of one.

The prohibition against the administration of 3% NaCl through a peripheral IV is due to the misconception that 3% NaCl can cause phlebitis and regional necrosis [21]. The notion that 3% NaCl can cause a local reaction is based on literature regarding the relationship between phlebitis and prolonged infusions of hypertonic total parenteral nutrition over days to weeks [22]. This literature is not applicable to either a bolus or short-term infusions of 3% NaCl used to acutely raise intracranial pressure and correct hyponatremia. It has been demonstrated that 3% NaCl does not cause a local reaction when administered through a peripheral vein in children [23,24]. Luu et al. retrospectively evaluated the administration of 3% NaCl during pediatric critical care transport [23]. One hundred and one children received a mean 3% NaCl bolus of 5.3 mL/kg over 47 min with 96% of doses administered via a peripheral line. One child received as much as 600 mL of 3% NaCl. No local reactions were noted. Similarly, Brenkert et al. retrospectively evaluated the administration of 3% NaCl in the pediatric emergency department [24]. Fifty-six patients received a median 3% NaCl bolus of 4.1 mL/kg over 17 min with 87% receiving the dose through a peripheral line. A 2-month-old received a 2 mL/kg bolus over 15 min through a 25-gauge catheter in the hand without complications. No local reactions were noted. Similar studies have been reported in adults [25]. Our group treated 71 episodes of hyponatremic encephalopathy in adults presenting to the emergency department with 500 mL of 3% NaCl over 6 h through a peripheral vein without local reactions or neurologic complications related to 3% NaCl [17]. A recent systematic review of nine studies that included 837 patients who received 3% NaCl through a peripheral vein failed to demonstrate an association with infusion-related adverse events [6].

Restricting the use of 3% NaCl to the intensive care unit can have unintended adverse consequences. The primary reason that a patient with hyponatremic encephalopathy should be transferred to an intensive care unit is not because of the monitoring required to administer 3% NaCl safely but rather the need to monitor the patient’s neurologic symptoms closely, as a patient with hyponatremic encephalopathy can have a respiratory arrest and may require intubation and ventilation [1]. It has been demonstrated in adults that 3% NaCl can be safely administered outside of the ICU in either the emergency department or medical floor without complications associated with the infusion [17,26]. The decision about transferring the patient to intensive care unit should be guided based on the degree of neurologic symptoms and on the length of time that the 3% NaCl infusion will be needed. There is a report of a 35-year-old adult with acute hospital-aggravated hyponatremia, where the physicians recommended treating with intravenous 3% NaCl, yet they had to resort to hourly oral sodium chloride tablets as their hospital prohibited the use of 3% NaCl in the non-ICU setting and there was a city-wide public health emergency preventing transfer to the ICU [27]. Pharmacy restrictions on 3% NaCl administration are not consistent with the lack of restrictions on hypertonic 20% mannitol (1100 mOsm/L) and 8.4% sodium bicarbonate (2000 mOsm/L), both of which have an osmolality greater than that of 3% NaCl (1027 mOsm/L).

We have previously recommended that 3% NaCl should only be administered in patients with symptomatic hyponatremia [1]. It is now recognized that even mild chronic hyponatremia in adults can result in subtle neurological impairment affecting both gait and attention [28], and that hyponatremia in the elderly is associated with falls and fractures [29]. Therefore, even patients with mild symptoms of hyponatremia may benefit from a partial correction of serum sodium with 3% NaCl. Three-percent NaCl should be able to be administered safely as a bolus or as a continuous infusion of limited duration in the non-ICU setting, provided that an electronic infusion smart pump with free-flow protection and dosing limits is used [30].

Cerebral demyelination is primarily a complication that occurs in patients with severe and chronic hyponatremia [31]. Numerous other risk factors contribute to this, including liver disease, hypoxia, hypokalemia, hypophosphatemia, malnutrition and thiazide diuretics [32,33]. What is considered a safe magnitude of correction is debatable, but both human and animal studies have demonstrated that a 25 mEq/L increase over 48 h can produce demyelinating lesions [32,34]. An overcorrection of hyponatremia is not so much related to the concentration of NaCl administered or the rate of administration, but more to the total quantity administered and the renal response. Hyponatremia typically results from excess vasopressin in conjunction with free water intake. When the stimulus for vasopressin abates, a free water diuresis will occur and the potential for an overcorrection exists independent of the type and quantity of fluid administered [35]. The indiscriminate use of a prolonged 3% NaCl infusion can result in overcorrection of hyponatremia regardless of the rate, route and setting of administration, and this should be avoided. We have proposed using intermittent 3% NaCl boluses in order to acutely raise serum sodium, while at the same time avoiding overcorrection [11], and this approach has been incorporated in the European Clinical Practice Guidelines [2]. For neurosurgical patients who may need a prolonged infusion of 3% NaCl to treat increased ICP, close monitoring in an ICU setting may be necessary. Similarly, patients with severe hyponatremia, Na < 115, may need close sodium monitoring in an ICU setting to prevent overcorrection, regardless of the type of fluid administered [35].

There are several limitations to this study. This study was not designed to evaluate the exact pharmacy protocols and guidelines for the administration of 3% NaCl. As such, we cannot conclude that the restrictions on the rate and volume of 3% NaCl may have prohibited a therapeutic dose of 3% NaCl for the treatment of hyponatremic encephalopathy or increased intracranial pressure. Similarly, we do not know if there were exceptions in the protocols for allowing 3% NaCl to be administered through a peripheral IV or in the non-ICU setting. The survey was also conducted in 2011 and there was a delay in publishing these results. These results may not reflect current children’s hospital pharmacy practices. The responses of this survey were likely representative of other children’s hospital pharmacies at that time, as the survey had a high response rate and represented a diverse group of children’s hospital pharmacies across the country. This survey was not sent out to treating physicians and does not reflect their view on the use of 3% NaCl.

## 5. Conclusions

The majority of children’s hospital pharmacies have policy and practice guidelines which restrict the use of 3% NaCl, yet they do not have similar guidelines restricting hypertonic mannitol and sodium bicarbonate. The most concerning of these restrictions are those prohibiting the administration of 3% NaCl through a peripheral vein and in the non-ICU setting. These restrictions could have deleterious consequences by delaying potentially life-saving therapy for symptomatic hyponatremia. While this study was restricted to children’s hospital pharmacies, similar barriers could exist in adult facilities. Current evidence supports that 3% NaCl can be administered safely through a peripheral IV and in the non-ICU setting. Children’s hospital pharmacies should review their policies and consider revising their restrictions on the administration of 3% NaCl.

## Figures and Tables

**Table 1:** Survey question related to 3% sodium chloride administration.

1	Do you have written policy/practice guidelines for the administration of 3% sodium chloride?
2	Is there a volume limit you will dispense?
3	Is there a limit on the rate of administration?
4	Is it approved for use on “acute” care units, i.e., non-ICU patients?
5	Do you allow peripheral IV administration of 3% sodium chloride?
6	If you allow peripheral IV administration of 3% sodium chloride, are there any restrictions?
7	Do you have written policy/practice guidelines for hypertonic sodium bicarbonate?
8	Do you have written policy/practice guidelines for hypertonic mannitol?

## Data Availability

The data presented in this study are openly available in The Harvard Dataverse Repositor at https://doi.org/10.7910/DVN/7J4B6P, (accessed on 17 October 2021).
